# AI-based digital pathology and spatial proteomics enable precision oncology: A case report of recurrent melanoma in a young patient

**DOI:** 10.1038/s41698-026-01569-w

**Published:** 2026-07-28

**Authors:** Jéssica Guedes, Nicole Woldmar, Eszter Baltas, András Kriston, Ede Migh, Ferenc Kovács, Henriett Oskolás, Matilda Marko-Varga, Roger Appelqvist, Elisabet Wieslander, A. Marcell Szasz, Zoltán Veréb, Rolland Gyulai, István Balázs Németh, Johan Malm, Peter Horvath, Jeovanis Gil, György Marko-Varga

**Affiliations:** 1 Section for Clinical Chemistry, Department of Translational Medicine, Lund University, Lund, Sweden; 2 Department of Dermatology and Allergology, Albert Szent-Györgyi Faculty of Medicine, University of Szeged, Szeged, Hungary; 3 Synthetic and Systems Biology Unit, HUN-REN Biological Research Centre (HUN-REN BRC), Szeged, Hungary; 4 Single-Cell Technologies Ltd., Szeged, Hungary; 5 Clinical Protein Science & Imaging, Biomedical Centre, Department of Biomedical Engineering, Lund University, Lund, Sweden; 6 Department of Internal Medicine and Oncology, Semmelweis University, Budapest, Hungary; 7 University Hospital Szeged Biobank, Szeged, Hungary; 8 Department of Dermatology, Venerology and Oncodermatology, Faculty of Medicine, University of Pécs, Pécs, Hungary; 9 Institute of AI for Health, Helmholtz Zentrum München, Neuherberg, Germany; 10 Institute for Molecular Medicine Finland, University of Helsinki, Helsinki, Finland; 11 Chemical Genomics Global Research Lab, Department of Biotechnology, College of Life Science and Biotechnology, Yonsei University, Seoul, Republic of Korea; 12 1st Department of Surgery, Tokyo Medical University, Tokyo, Japan

**Keywords:** Biomarkers, Cancer, Computational biology and bioinformatics, Oncology

## Abstract

Metastatic melanoma presents clinical challenges due to tumor heterogeneity and treatment resistance. Here, we report an integrative workflow combining AI-based digital pathology with spatial proteomics to support personalized treatment strategies in a case of a young patient with recurrent melanoma and multiple metastases. Our AI model trained on H&E images identified two spatially separated cell subpopulations (PT1 and PT2) within the primary lesion, along with metastatic areas and stromal components. MS-based proteomics was used to map the spatial proteome across the clinically relevant regions. Our findings indicate inter-tumor heterogeneity and increased kinases associated with target-drug resistance. Convergent morphological and proteomic signatures identified PT1 as an aggressive melanoma subtype and the likely metastatic driver. Augmented glycolytic signaling and mitochondrial metabolism were identified as drivers of melanoma progression in this patient. Our findings suggest that targeted therapies may provide limited benefit, while the combination with metabolic inhibitors could represent a more effective treatment option for the patient.

## Introduction

Melanoma, although less prevalent than other skin cancers, accounts for the majority of skin cancer–related deaths owing to its high metastatic potential and pronounced inter- and intratumor heterogeneity^1,2^. In adolescents and young adults, it is uncommon, yet when it occurs, the clinical course can be aggressive and unpredictable^3,4^. Metastatic spread remains the principal cause of mortality; once dissemination occurs, prognosis worsens and treatment efficacy declines^5–7^. A persistent challenge in advanced disease is pinpointing the metastasis-initiating and therapy-resistant subclones within the native tissue architecture^8^. Conventional histopathology and bulk genomic assays often underrepresent spatial heterogeneity and may not consider minority populations that seed relapse.

Recent advances enable interrogation of tumor biology in situ. Artificial-intelligence (AI)–based digital pathology can detect subtle, prognostically relevant morphologic patterns and resolve distinct cellular phenotypes on routine hematoxylin–eosin (H&E) slides^9,10^, while spatial proteomics profiles region-specific protein expression and signaling states, anchoring molecular data to histology^11,12^.

The digital pathology field has advanced rapidly toward sophisticated frameworks capable of quantifying spatial relationships between cells and capturing tissue architectural complexity. New models enable reproducible cell-to-cell spatial analysis of whole-slide images, generating a broader array of interpretable pathology features and empowering researchers to study cell-to-cell spatial relationships at scale^13^. Building on this, hybrid frameworks such as SlideMamba^14^ integrate local and global morphological information in whole-slide images through entropy-guided adaptive fusion. In cancer research, deep learning-based analysis of tumor-associated stroma from routine H&E slides has demonstrated significant prognostic value for disease-specific survival^15^ and recurrence, particularly when combined with spatially resolved molecular methods^12^.

**Fig. 1:**
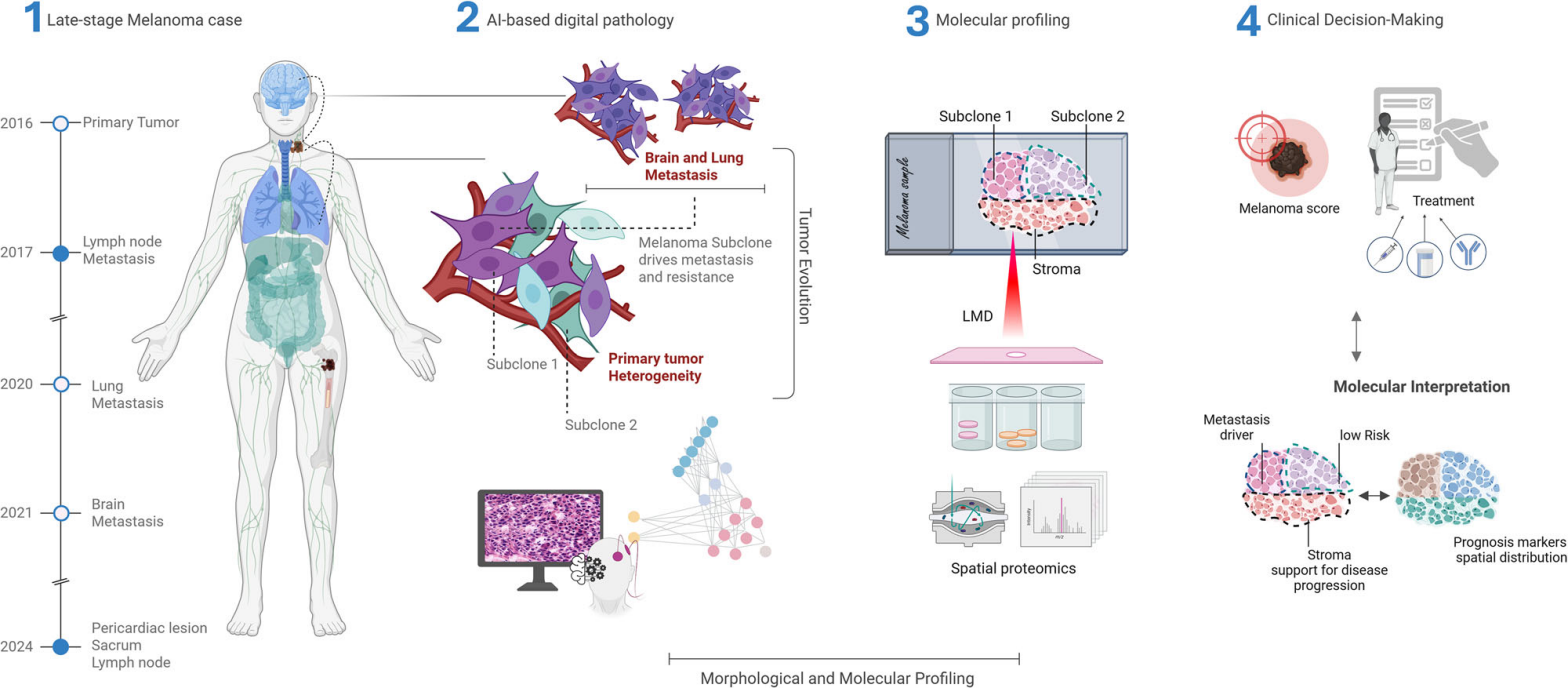
Overview of study design and workflow. **1** Clinical timeline of a recurrent melanoma patient over 8 years. **2** Histopathological assessment integrated with AI-assisted image analysis to identify distinct tumor populations and stromal components. **3** Spatial proteomics performed on laser-microdissected areas, guided by AI-based region selection. **4** Biological interpretation of the proteomic data to characterize molecular features and inform potential treatment strategies in this clinical case.

Here, we apply an integrated workflow that couples AI-guided histopathologic analysis with laser-microdissection–based spatial proteomics in a young patient with recurrent metastatic melanoma (Fig. 1). By aligning computationally defined subpopulations with region-resolved proteomes across the primary tumor and distant metastases, we map clonal heterogeneity and evolutionary change over an eight-year disease course, linking molecular profiles to clinical events. This case illustrates how spatial, multimodal profiling can identify metastasis-competent populations and reveal actionable vulnerabilities, offering hypothesis-generating guidance for individualized treatment beyond conventional diagnostics.

## Results

### Case presentation and medical history

A 24-year-old woman was diagnosed in 2016 with cutaneous melanoma on the occipital scalp. The primary tumor measured a Breslow thickness of 3.04 mm and was classified as pT3a. She underwent a wide local excision with a sentinel lymph node biopsy at our melanoma center. The sentinel node was negative, and in the absence of further dissemination, the disease was initially staged as IIA, and adjuvant low-dose interferon alpha-2b was started (Fig. 1).

Approximately six months after starting interferon treatment, the patient noted a new palpable lymph node in the right cervical (neck) region. Complete regional lymph node dissection confirmed melanoma metastasis in 1 of 14 nodes, consistent with regional recurrence (stage III). No systemic therapy was initiated, and she was followed under close surveillance. Three years later, a routine PET-CT revealed a solitary 2 cm lesion in the right lung. In 2020, she underwent video-assisted thoracoscopic lobectomy, and histopathology confirmed a melanoma metastasis. She then received one year of adjuvant targeted therapy with a combined BRAF and MEK inhibitor.

Three months after completing targeted therapy, she presented with a generalized epileptic seizure. Brain MRI demonstrated multiple intracranial metastases. Stereotactic radiosurgery was performed on the largest lesions, followed by initiation of immune checkpoint blockade (ipilimumab plus nivolumab). Treatment was discontinued after the first cycle due to grade III immune-related hepatitis, which required high-dose corticosteroids. Rapid progression of symptomatic brain metastases ensued. She subsequently underwent whole-brain radiotherapy (WBRT) with simultaneous integrated boost to dominant lesions, combined with temozolomide chemotherapy. A refractory metastasis in the left temporal lobe was surgically resected, providing symptomatic relief and tissue for analysis. After neurosurgical recovery, BRAF and MEK inhibitor therapy was re-initiated.

Over the next two years, the patient’s condition stabilized and improved under resumed targeted therapy. During the third year, oligometastatic recurrences were managed with focal stereotactic body radiation therapy (SBRT) to mediastinal and subcarinal lymph nodes, the sacrum, and a pericardial nodule. In the final months of life, she developed increasingly frequent seizures, including status epilepticus, which significantly impaired her quality of life. Notably, restaging evaluations during this period showed no evidence of new extracranial progression, suggesting neurological decline was primarily attributable to severe epilepsy and/or microscopic residual disease in the brain. Despite intensive antiepileptic therapy, her neurological condition deteriorated rapidly, and she died in 2024, approximately eight years after her initial melanoma diagnosis.

### Investigations and analytical workflow

In light of the patient’s disease course with multiple recurrences, we conducted a comprehensive integrative analysis of available tumor specimens to better characterize the disease biology. Archival formalin-fixed, paraffin-embedded (FFPE) tissue blocks were obtained from the primary tumor, three distinct lung metastases, and one brain metastasis resected during the clinical course. We first applied a supervised AI-based digital pathology approach to the H&E-stained tissue sections from the primary melanoma and selected metastases. Using deep learning algorithms for single-cell image segmentation and classification, the model was trained to recognize and label different histological components, including melanoma cell subpopulations, melanoma in situ cells, stromal regions (e.g., areas rich in tumor-infiltrating lymphocytes, TILs), and normal tissue elements, directly on the digitized slide images. This allowed us to map the spatial distribution of distinct tumor cell populations and microenvironment features within each tissue section at single-cell resolution. The AI model’s performance was validated on held-out image regions, and it achieved high classification accuracy for the identified classes.

Guided by the AI-generated tissue maps, we then performed laser capture microdissection to isolate specific regions of interest from the FFPE sections for spatially resolved proteomic analysis. In the primary tumor, we microdissected representatives of the two distinct melanoma cell subpopulations identified by the AI (hereafter referred to as PT1 and PT2). Likewise, from the metastasis specimens, we isolated tumor cell–rich regions (predominantly corresponding to the PT1-like areas as identified by the AI, see Results) as well as portions of the surrounding stromal tissue (both with and without heavy TIL infiltration). A total of five microdissected sample categories were collected: PT1 cells (primary tumor), PT2 cells (primary tumor), tumor cells from a lung metastasis, tumor cells from the brain metastasis, and primary tumor stroma. In parallel, to capture broader proteomic differences, we also analyzed bulk (whole-tissue) sections from each tumor site. The microdissected material (on the order of a few thousand cells per sample) and bulk tissue lysates were processed for quantitative proteomics using high-resolution mass-spectrometry workflows optimized for FFPE samples^16,17^. The proteomic datasets were then analyzed with a multi-pronged computational approach: we performed unsupervised clustering to examine sample-to-sample molecular relationships, and conducted pathway enrichment analyses (including one-dimensional and two-dimensional enrichment) to identify signaling pathways and biological processes that were up- or down-regulated in metastases relative to the primary tumor. We also carried out a connectivity map analysis, correlating the tumor’s proteomic signature with drug response databases (L1000-based profiles), in order to predict potential candidate therapeutic compounds or mechanisms of action that might be effective against this tumor. Finally, to put the patient’s tumor microenvironment findings in context, we compared the proteomic profile of her primary tumor stroma to stroma-focused proteomic profiles from an external reference cohort of melanoma patients with known outcomes (non-recurrent vs. recurrent disease^12^).

### AI-based digital pathology identifies distinct tumor subpopulations linked to metastasis

**Fig. 2:**
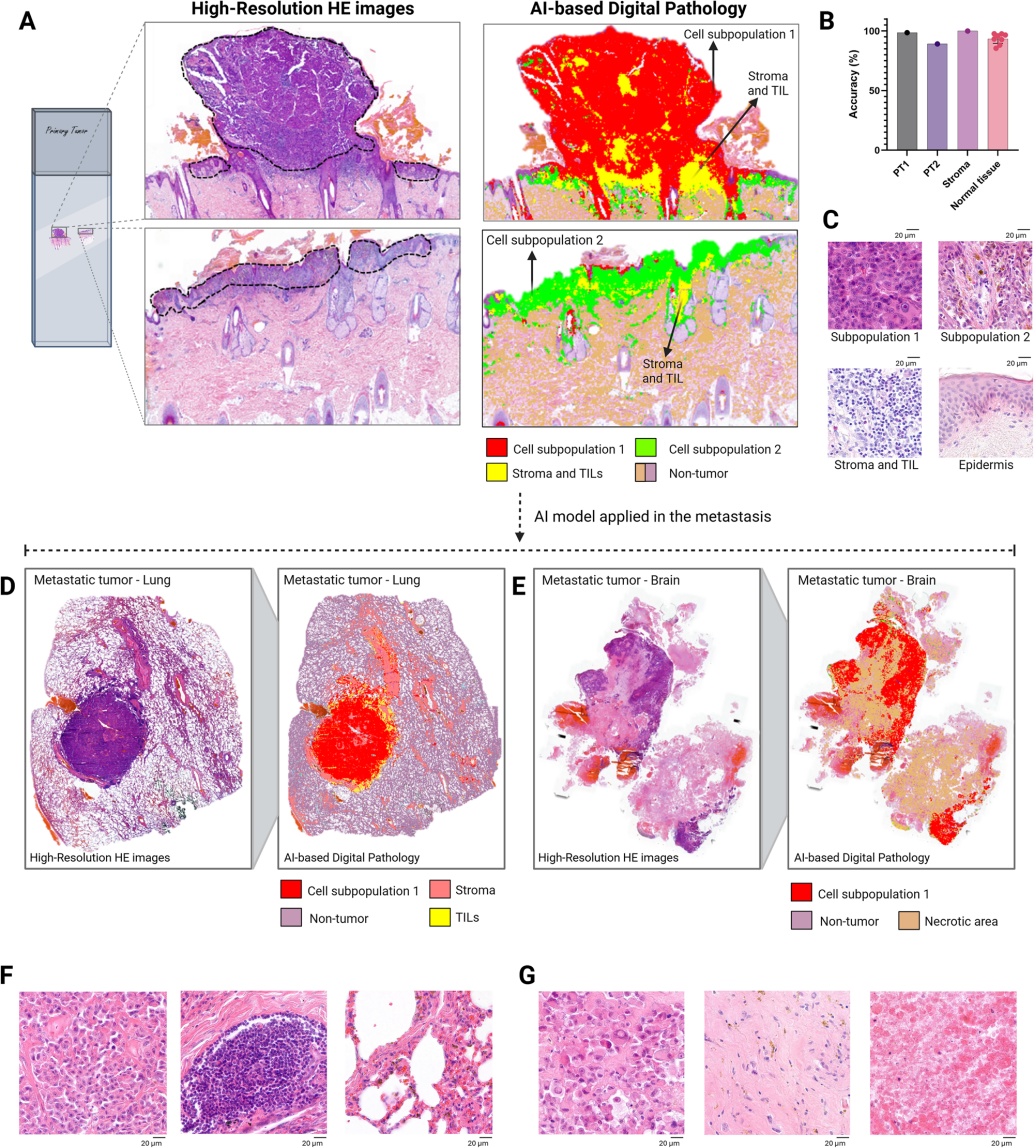
AI-based digital pathology approach - representative image prediction. **A** Deep-learning–based image segmentation and classification using the BIAS software identified two spatially distinct melanoma subpopulations within the primary tumor: PT1 (red) and PT2 (green), as well as stroma/TIL (bright yellow), and non-tumor regions (beige and purple). **B** Overall prediction accuracy (%) of the AI model trained on the primary tumor. **C** High-magnification (40×) H&E-stained images from the primary tumor showing representative areas of PT1, PT2, stroma/TILs, and normal epidermis. **D** AI model applied to lung metastasis tissue, AI-prediction of tumor (red), stroma/non-tumor (light pink), TILs (yellow), non-tumor regions (purple). **E** AI model applied to brain metastasis, AI-prediction of tumor (red), stroma and necrotic area (beige), non-tumor regions (purple). **F** H&E-stained lung metastasis images (40×) illustrating tumor cells (left), stroma/TIL-rich (center), and normal tissue (right) **G** H&E-stained brain metastasis images (40×) showing tumor cells (left), stroma area (center), necrotic area (right).

Applying the AI-driven image analysis to the primary tumor slide revealed two spatially segregated melanoma cell subpopulations within the primary lesion, which we designate PT1 and PT2 (Fig. 2A–C). The AI model’s tissue segmentation map showed PT1 cells (colored red in the model output) concentrated in certain regions of the primary tumor, whereas PT2 cells (colored green) occupied different regions (Fig. 2A). In addition, the model annotated stromal/TIL-rich areas (yellow) and normal tissue components such as epidermis, dermis, and skin adnexal structures (shown in other colors). The AI classifier demonstrated high performance in distinguishing these classes, with an accuracy of approximately 99% for PT1 and 89% for PT2, and an overall prediction accuracy of 94% in the primary tumor (Fig. 2B, and Supplementary Data Fig. S1). These results indicate the model could capture subtle morphological differences between the two melanoma cell subpopulations and other tissue elements.

After training, a total of 171,875 cells were detected and classified, of which 57,641 belonged to PT1 (mean confidence: 94.86%), 26,957 to PT2 (mean confidence: 86.86%), 23,524 were lymphocytes (mean confidence: 89.94%), and 63,753 were classified as other cells (*e.g*., epidermis, dermis, and skin adnexal structures). Restricting the analysis to cells with a confidence threshold of ≥99%, significant differences in nuclear and cell size were observed across cell types. PT2 cells had a mean nuclear area comparable to other cells found in the tissue (34.6 µm² and 35.1 µm², respectively), whereas PT1 cells displayed a significantly larger mean nuclear area of 50.5 µm² (Supplementary Data Fig. S2 and Tables S1, S2). This cell type mapping indicates notable intratumor heterogeneity in the primary melanoma.

We next applied the same pretrained AI model to histological images from the resected metastases of the patient. To assess the generalization capability of the original model beyond its primary training domain, experts manually annotated the tumor regions from brain and lung tissue; the skin model was subsequently validated against annotated datasets from brain and lung tissue. Remarkably, in both the lung and brain metastatic samples, the model identified tumor cell populations whose morphology closely matched the PT1 subpopulation from the primary tumor. In the lung metastasis, virtually all tumor cells were classified as PT1-like (red), intermingled with some regions of stromal tissue and infiltrating lymphocytes (yellow), and adjacent normal lung parenchyma at the periphery (Fig. 2D and Supplementary Data Table S3). In the brain metastasis, the model likewise predominantly detected PT1-like tumor cells (red), alongside areas of necrosis (beige) and sparse stromal or immune infiltrate (Fig. 2E and Supplementary Data Table S3). These consistent findings across two anatomically distant metastases suggest that the PT1 subclone from the primary tumor gave rise to the metastatic lesions. In contrast, cells corresponding to the PT2 phenotype (green) were essentially absent from the metastases. Notably, the brain metastasis tissue showed extensive necrotic zones and minimal lymphocytic infiltration compared to the lung metastasis, underscoring how the tumor microenvironment differed by metastatic site; yet even in the brain, the viable tumor cells present were identified as PT1. Taken together, the AI-guided histopathology results revealed early divergence of two melanoma subpopulations in the primary tumor and implicated the PT1 subpopulation as the likely metastasis-initiating clone in this patient. These insights provided a spatial map of tumor heterogeneity to guide downstream molecular analyses. Guided by the AI findings, we proceeded to isolate the PT1 and PT2 regions from the primary tumor, as well as tumor cell regions from the metastases, for comparative proteomic profiling.

### Spatial proteomics reveals metabolic reprogramming and therapeutic vulnerabilities

**Fig. 3:**
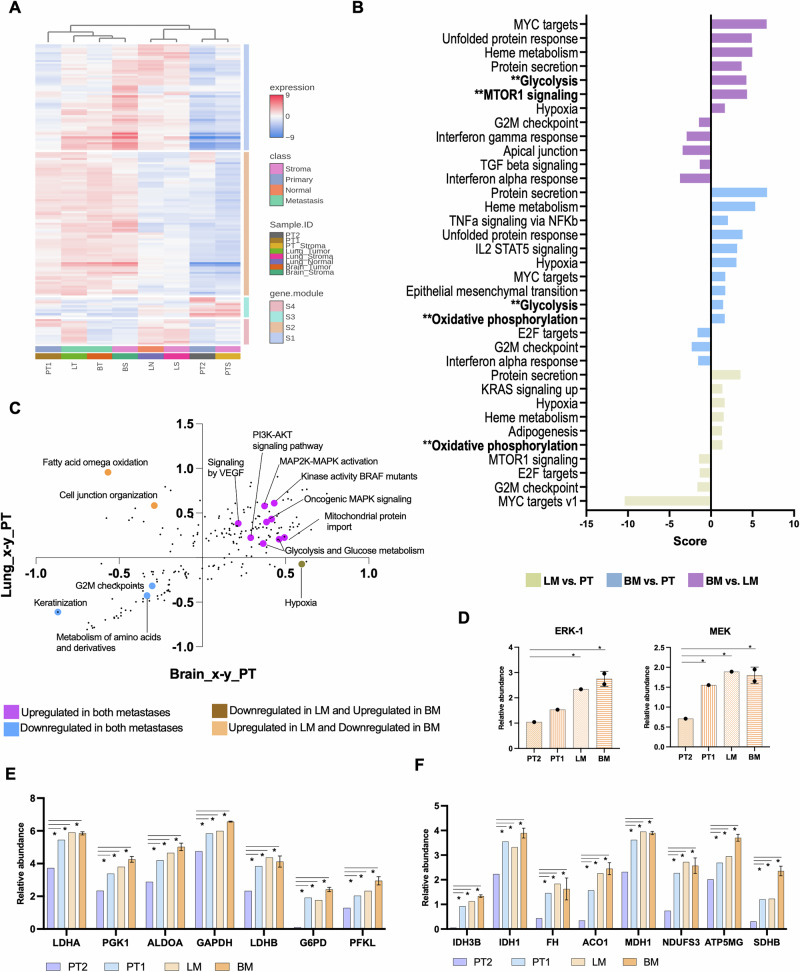
Proteome profiling of primary tumor and distant metastasis. **A** Hierarchical clustering of samples based on global proteomic profiles, illustrating distinct molecular differences between the two primary tumor subpopulations (PT1 and PT2), the lung (LM) and brain (BM) metastases, stromal regions (lung stroma, LS; primary tumor stroma, PTS; brain stroma, BS), and normal lung tissue. **B** 1D-enrichment analysis of whole-tissue proteomic data, showing significantly enriched pathways (*p* < 0.05). **C** 2D-enrichment pathway analysis. Pathways are color-coded by behavior across the two metastases: purple indicates upregulation in both; blue, downregulation in both; brown, downregulation in LM but upregulation in BM; and light orange, upregulation in LM but downregulation in BM (*p*-value < 0.05). **D** Relative protein abundance of MAPK pathway components MEK and ERK1. **E** Relative abundance of proteins involved in glycolysis. **F** Relative abundance of proteins involved in the TCA cycle (IDH3B, IDH1, FH, ACO1, MDH1) and oxidative phosphorylation (OXPHOS: NDUFS3, ATP5MG, SDHB). *Log2foldchange > 1.

Proteomic analysis of microdissected samples and whole-tissue extracts identified over 6,000 proteins across the primary and metastatic tumor regions. Unsupervised hierarchical clustering of global proteome profiles showed a separation between the primary tumor and metastatic samples, suggesting inter-tumor heterogeneity (Fig. 3A). Intriguingly, the two subpopulations from the primary tumor (PT1 and PT2) also clustered apart from each other, which may reflect the intra-tumor heterogeneity suspected from the histology. The PT1 proteomic profile showed greater similarity to the lung and brain metastases, whereas PT2 displayed a distinct proteome signature that was less similar to the metastases. This molecular pattern may imply that the PT1 subclone in the primary tumor was responsible for seeding the distant metastases. In contrast, PT2 appears to represent a divergent tumor cell population with a less aggressive phenotype that may not have significantly contributed to metastatic spread.

To identify key pathways associated with the metastatic progression in this case, we performed pathway enrichment analyses on the proteomic data. A 1D-enrichment analysis (focused on a curated set of cancer-related hallmarks) revealed a significant upregulation of glycolysis and oxidative phosphorylation (OXPHOS) pathways in the metastases compared to the primary tumor (*p* < 0.05 for enrichment of both metabolic pathways) (Fig. 3B). This suggests that the metastatic tumors may have elevated activity in both aerobic glycolysis and mitochondrial energy production. Such dual metabolic upregulation could indicate a high degree of metabolic plasticity, a feature that has been associated with more aggressive tumor behavior and the capacity to thrive in diverse microenvironments. A complementary 2D-enrichment analysis further highlighted dysregulated signaling processes accompanying metastasis. Notably, MAPK pathway signaling was significantly enriched (*p* < 0.05) in the metastatic lesions relative to the primary, along with related downstream signaling cascades such as the PI3K–AKT–mTOR pathway, while markers of anti-tumor immune response (for example, PD-1 signaling) were downregulated (*p*-value < 0.05) in the metastases (Fig. 3C). At the individual protein level, the metastatic tumors showed higher abundance of multiple glycolytic enzymes and mitochondrial proteins (Log2FC ≥ 1) (including components of the tricarboxylic acid cycle and electron transport chain), as well as increased levels of key signaling proteins like AKT and mTOR, compared to the primary tumor (Supplementary Data Fig. S3). These findings suggest that, in this patient, metastatic progression was potentially driven by a coordinated metabolic and signaling reprogramming: the tumor cells in metastases adapted by boosting energy-production pathways and activating growth and survival signaling networks.

Spatially resolved proteomic comparisons between the PT1 and PT2 subpopulations provided additional insights. Subpopulation PT1 (the potential metastasis-prone clone) exhibited a higher abundance of numerous mitochondrial and glycolytic proteins even within the primary tumor, relative to subpopulation PT2 (Fig. 3E–3F). This metabolic signature in PT1 was more pronounced at metastatic sites, consistent with PT1 being a potential source of those metastases. Meanwhile, PT2 showed a comparatively lower abundance of those metabolic proteins and lacked the upregulation of MAPK pathway components seen in PT1 and in metastases. At the protein level, several components of the MAPK pathway appeared to be more abundant in the metastatic tumor regions compared to the primary tumor; for example, MEK1/2 (MAP2K2), ERK1 (MAPK3), and the small GTPase RRAS were among the proteins showing higher abundance values in the metastases (Log2FC≥1) (Fig. 3D). PT1 in the primary tumor also showed higher MAPK pathway protein levels than PT2, though not as high as the overt metastases. In summary, the spatial proteomics results suggest that the subclone PT1 was characterized by a more “aggressive” molecular program, marked by enhanced glycolytic and mitochondrial metabolism and activation of proliferative signaling pathways, even at the time of the primary tumor, whereas the other subclone (PT2) had a different, potentially less aggressive profile.

It is important to note that, given the limited sample size of this single-patient analysis, the molecular results are presented as exploratory and hypothesis-generating.

### Proteomic profiling and insights for off-target treatment strategies

In addition to common oncogenic pathways such as MAPK, the proteomic data raised the possibility of therapy resistance mechanisms in the metastatic tumor regions. Notably, YES1 and SRC showed higher abundance in the metastatic tissues compared to the primary tumor (Supplementary Data Fig. S4). These kinases have been implicated in resistance to BRAF/MEK inhibitors via noncanonical activation of ERK signaling^18^. The presence of elevated SRC/YES1 in our patient’s metastases suggests that even if MAPK pathway inhibitors (BRAF/MEK inhibitors) initially have an effect, the tumor may have the molecular toolkit to bypass MAPK blockade through alternate routes, potentially leading to drug resistance. Indeed, the patient did experience disease relapse after adjuvant BRAF/MEK inhibitor therapy, which could be consistent with the emergence of such resistance mechanisms. These findings may imply that while BRAF–MEK targeted therapy was a relevant treatment approach (given the MAPK activation in the tumor), it likely needed to be complemented by additional strategies to prevent or overcome resistance.

One attractive therapeutic strategy emerging from our analysis is targeting the tumor’s metabolic vulnerabilities. The concurrent elevated levels of proteins from glycolysis and OXPHOS in the metastases suggest the tumor cells may rely on robust metabolic flexibility. Agents that disrupt cancer cell metabolism could therefore be beneficial adjuncts. In this regard, metformin, a widely used oral antidiabetic drug, stands out as a candidate for combination therapy. Beyond its glucose-lowering effects, metformin is known to inhibit mitochondrial complex I, thereby reducing OXPHOS, and it modulates several cellular pathways linked to cancer cell apoptosis and metastatic progression^19–21^. Metformin has a well-characterized safety profile and, importantly, has been studied as an adjuvant treatment in various cancers, including melanoma^22–24^. Preclinical and clinical evidence suggest that adding metformin can enhance the efficacy of immune and targeted therapies, possibly by hampering tumor metabolic adaptation and alleviating therapy-induced tumor hypoxia^22,23^. Retrospective analyses have even found that diabetic melanoma patients on metformin had improved survival outcomes and lower rates of brain metastasis compared to those not on metformin^24^. Given our patient’s tumor showed elevated abundance of OXPHOS proteins, integrating a mitochondrial inhibitor like metformin with standard therapy might have been a reasonable approach to explore, potentially slowing tumor progression or resensitizing the tumor to other treatments.

**Fig. 4:**
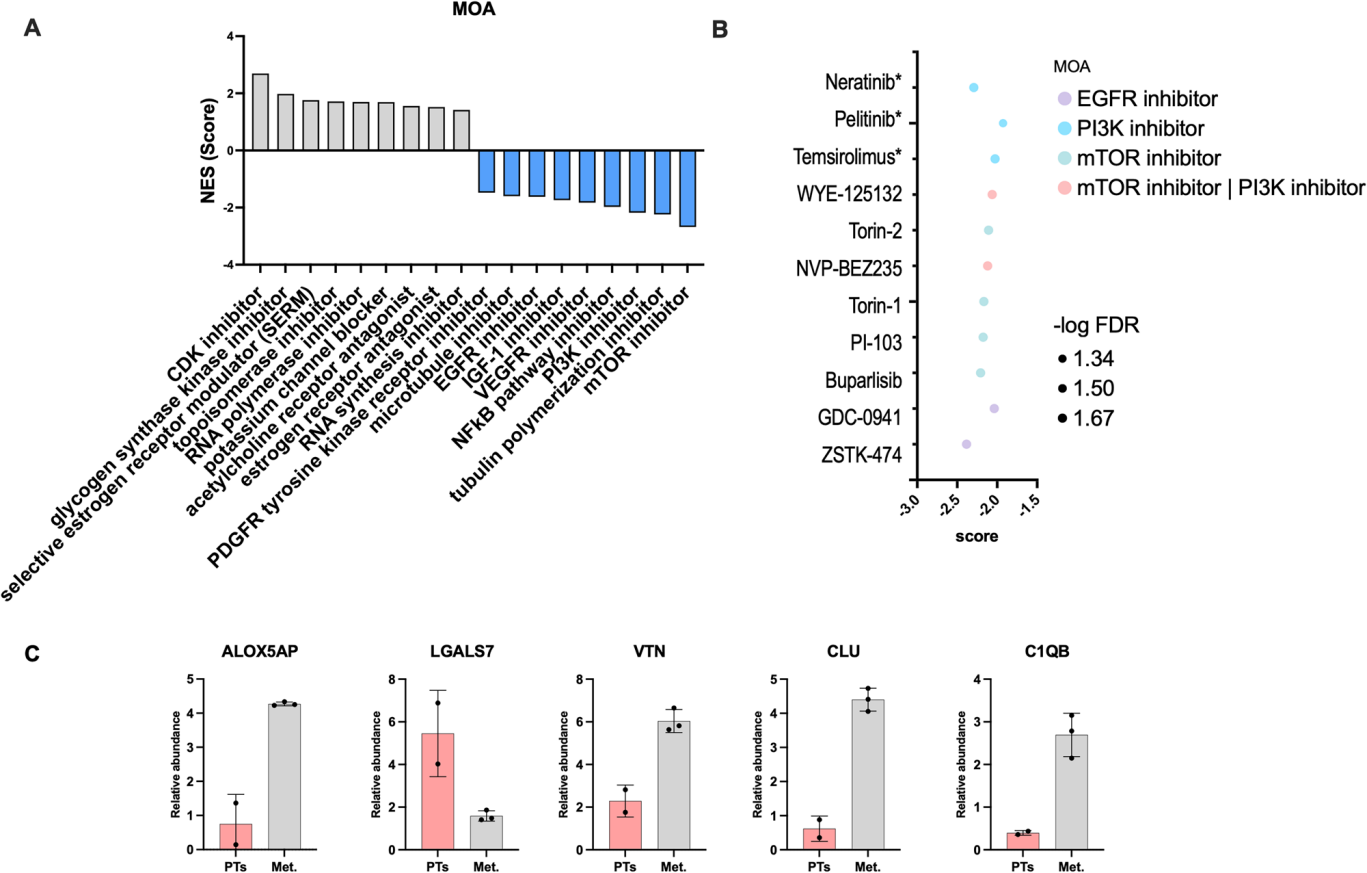
Drug connectivity and biomarker selection analyses. **A** Enrichment analysis of drug mechanisms of action, highlighting pathways with potential therapeutic relevance in this case. **B** inhibitor compounds with statistically significant enrichment scores (FDR < 0.05). *FDA-approved drugs. **C** Top five proteins identified through biomarker selection analysis, integrating multiple machine learning approaches to pinpoint features with the strongest discriminative power across tumor subpopulations and metastatic sites.

To systematically identify additional therapeutic opportunities, we performed a drug connectivity analysis using our proteomic data. By correlating the tumor’s proteome signature with large-scale drug response databases, this analysis predicted several classes of targeted agents that could counteract the observed molecular profile. In particular, inhibitors of mTOR/PI3K signaling, EGFR, VEGFR, and agents targeting microtubule dynamics (tubulin polymerization inhibitors) were among the top candidates, showing strong negative enrichment scores (NES < –1.8) when matched to our tumor’s proteomic signature (Fig. 4A). These findings are consistent with our pathway-level observations, suggesting that AKT–mTOR and related proliferative signaling pathways may have been active in the metastatic tumor, potentially representing candidates for therapeutic targeting in future studies. Drilling down to specific compounds, the connectivity map identified eleven individual drugs with statistically significant scores (FDR < 0.05) for potential efficacy. Notably, this list included two EGFR inhibitors (neratinib and pelitinib) and one mTOR inhibitor (temsirolimus) that are already FDA-approved for cancer treatment (Fig. 4B). Although EGFR inhibitors have historically shown limited single-agent activity in melanoma, there is growing evidence that they can be beneficial in combination regimens or in certain molecular contexts^25^. Indeed, combining EGFR blockade with BRAF/MEK inhibitors has yielded encouraging results in preclinical models and early trials, by helping to overcome or delay resistance to BRAF-targeted therapy^26–29^. Among the EGFR inhibitors identified, pelitinib (an irreversible EGFR tyrosine kinase inhibitor) emerged with a strong predicted benefit in our analysis. Pelitinib has demonstrated superior anti-tumor efficacy compared to some other EGFR inhibitors in preclinical studies of BRAF-mutant melanoma, including suppressing cell migration and metastatic colonization when used alongside BRAF inhibition^28^. This raises the possibility that adding an EGFR inhibitor like pelitinib to the patient’s treatment might have enhanced the response or mitigated resistance, especially given the SRC/YES1-mediated ERK reactivation noted in the tumor. Likewise, the identification of an mTOR inhibitor (temsirolimus) aligns with the tumor’s upregulated AKT–mTOR signaling, suggesting another rational combination partner to explore. Overall, these proteomics-driven insights point toward a multi-targeted treatment strategy: the tumor’s profile supports continuing MAPK pathway inhibition (BRAF/MEK inhibitors) but also indicates that co-targeting metabolic pathways (*e.g*., with metformin) and certain growth factor or survival pathways (EGFR, PI3K/mTOR) might yield a more durable and effective response for a tumor of this biology.

As part of our analysis, we also sought to identify individual protein biomarkers that distinguished the primary tumor from metastases. Using a machine-learning–based feature selection approach (incorporating algorithms such as sparse partial least squares, elastic net regression, random forests, and gradient boosting), we ranked proteins by their importance in differentiating primary vs. metastatic lesions. This integrative analysis highlighted five proteins as top candidates: clusterin (CLU), vitronectin (VTN), galectin-7 (LGALS7), arachidonate 5-lipoxygenase activating protein (ALOX5AP), and complement C1q subcomponent subunit B (C1QB) (Fig. 4C). Several of these proteins have known roles in cancer-related processes. For instance, CLU and VTN are involved in extracellular matrix interactions and cell survival; galectin-7 can modulate immune responses and apoptosis; ALOX5AP is linked to inflammatory pathways; and C1QB is part of the complement system, influencing inflammation and immunity. In fact, dysregulation of the tumor immune microenvironment and extracellular matrix remodeling were recurring themes in our pathway analyses. The over- or under-expression of these proteins in our patient’s metastases (relative to the primary tumor) might therefore reflect key alterations driving disease progression. While these five biomarkers require further validation in larger cohorts, they could serve as indicators of aggressive disease or even as potential therapeutic targets. For example, CLU and VTN have been studied as factors in melanoma metastasis and therapy resistance, and galectin-7’s role in immune evasion could make it a target for combination with immunotherapies. Identifying such proteins helps build a panel of molecules that merit closer investigation for their contribution to melanoma progression in young patients like ours.

In summary, the multi-regional proteomics approach suggests that this patient’s metastatic melanoma may present a shift toward a metabolism-fueled, signal-transduction-activated phenotype (glycolysis/OXPHOS and MAPK/AKT/mTOR upregulation). These molecular features both suggest a biological explanation for the aggressive clinical behavior and point to therapeutic avenues (*e.g*., metabolic modifiers and multi-pathway inhibitors) that might be explored to manage similar cases. The combination of AI-guided histology and high-resolution proteomics provided a detailed molecular map linking specific tumor subclones to distinct phenotypic traits and treatment vulnerabilities. It is important to note that the drug candidates identified through the drug connectivity analysis are based on molecular signature similarity and are intended as hypothesis-generating findings only.

### Stromal proteomic profile reflects an aggressive melanoma microenvironment

Beyond tumor cells themselves, the tumor microenvironment, particularly the stromal compartment, plays a crucial role in modulating melanoma progression and recurrence risk^12^. To investigate the patient’s tumor stroma in context, we compared the proteomic profile of her primary tumor’s stroma to stroma-focused proteomic data from two reference groups of early-stage melanoma patients: one group consisted of patients who remained disease-free long term (non-recurrent group), and the other group consisted of patients who developed melanoma recurrence within 3–5 years of initial treatment (recurrent group)^12^. This comparison aimed to see whether our patient’s stromal microenvironment more closely resembled that of indolent cases or aggressive ones.

**Fig. 5:**
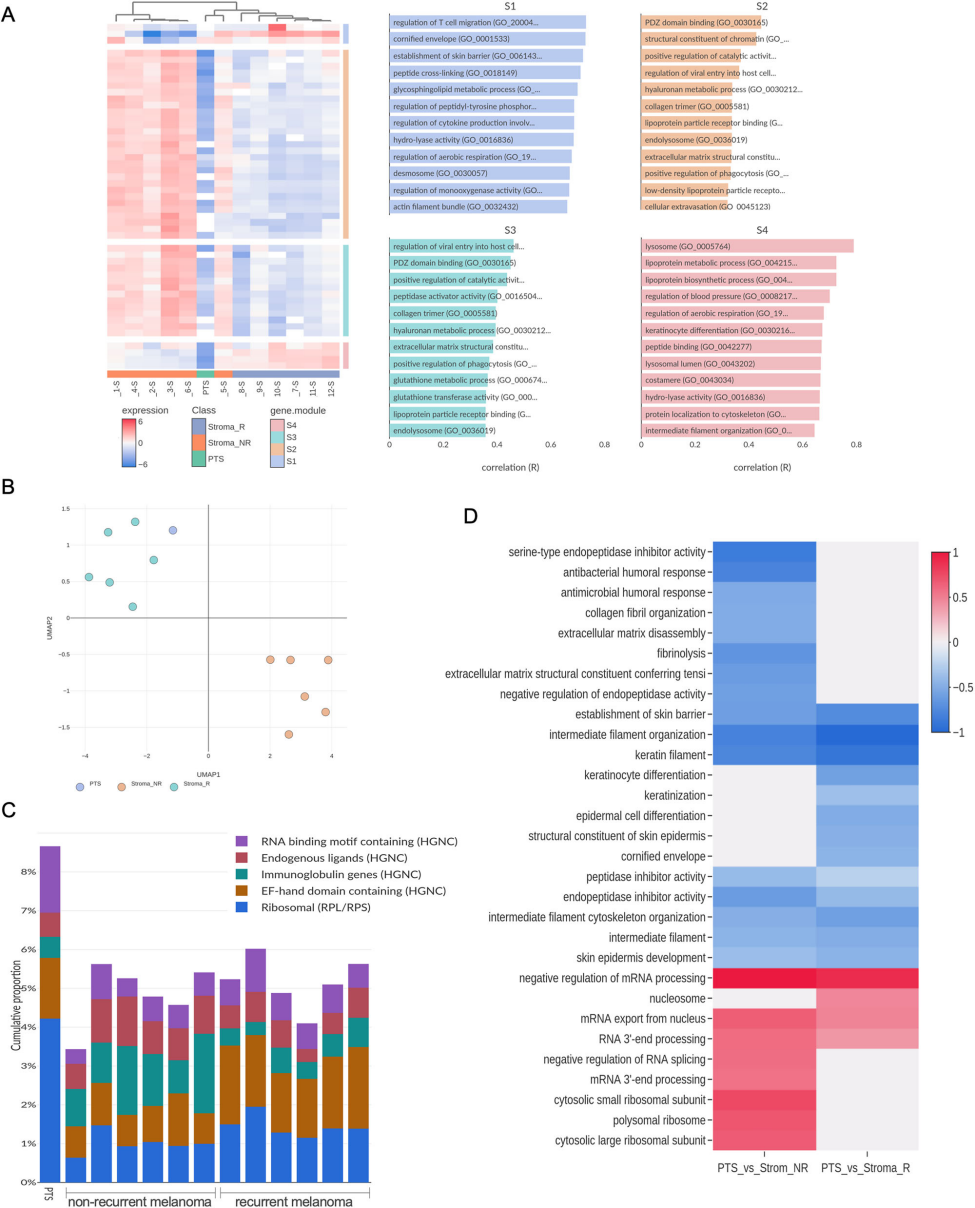
Proteomic profiling of the primary tumor stroma compared to stroma from non-recurrent and recurrent melanoma cases. **A** Hierarchical clustering based on spatially resolved proteomic data from the top 50 proteins with the highest standard deviation across all the samples, showing that the stroma from the target patient’s primary tumor (PTS) shares molecular similarity with stroma from recurrent and more aggressive melanoma cases (Stroma-R). Highlighted pathways reflect key biological processes enriched within four protein expression modules. **B** UMAP (Uniform Manifold Approximation and Projection) with at least 70% valid values across samples was included; missing values were imputed. **C** Comparison of protein composition across PTS, recurrent (Stroma-R), and non-recurrent (Stroma-NR) samples, illustrating differences in the distribution of protein types. **D** Gene Ontology (GO) pathway enrichment analysis highlighting biological processes significantly altered in the PTS stroma relative to the comparative groups.

Unsupervised clustering of the combined stromal proteomic data on the top divergent proteins (our patient’s stroma plus the two reference cohorts) revealed several distinct modules of co-expressed proteins (Fig. 5A). Strikingly, the proteomic profile of our patient’s stroma clustered tightly with the profiles from the recurrent melanoma stroma group, and was quite distant from the non-recurrent group. In particular, the patient’s stromal sample and the recurrent-case samples shared a characteristic downregulation of a set of proteins (visible as a cluster of under-expressed proteins in Fig. 5A, Module 2 and 3) involved in certain extracellular matrix and immune system pathways. Pathway enrichment analysis of these co-downregulated proteins indicated a significant suppression of biological processes related to extracellular matrix (ECM) organization and immune response activation in the stroma of our patient and the recurrent cohort, compared to the stroma of non-recurrent patients. These processes, including robust ECM maintenance and active local immune surveillance, have been linked in prior studies to better control of melanoma and lower risk of relapse^12^. The fact that our patient’s stromal proteome showed diminished activity in those pathways is consistent with her clinical course of multiple recurrences.

Uniform Manifold Approximation and Projection (UMAP) provided a complementary data visualization: the primary tumor stroma from our patient projected adjacent to the recurrent-group stromal samples in UMAP space, and visually distinct from the non-recurrent stromal cluster (Fig. 5B). As UMAP is an unsupervised dimensionality reduction method and does not provide statistical inference, this observation should be interpreted as descriptive; nonetheless, it suggests a similarity in stromal proteomic expression patterns between our patient’s aggressive case and those with recurrent disease in the reference cohort. Additionally, we examined the composition of the stromal proteome in terms of functional protein categories. Notably, the abundance of immunoglobulin-related proteins (e.g., various antibodies or B-cell–produced proteins) was markedly lower in our patient’s stroma (and in the recurrent group) compared to the non-recurrent group (Fig. 5C). Non-recurrent patients’ stroma showed a higher proportion of these immune-related proteins, which likely reflects a more vigorous presence of B cells or plasma cells and an active humoral immune response in the tumor microenvironment, potentially contributing to tumor containment. In our patient, the relative absence of such immune proteins in the stroma aligns with an immunologically “cold” microenvironment that might permit tumor escape.

Gene Ontology enrichment analyses corroborated these findings: the stroma of our patient (and the recurrent cohort) had consistent downregulation of pathways related to immune function (*e.g*., antigen presentation, lymphocyte activation) and ECM remodeling when compared to stroma from non-recurrent melanomas (Fig. 5D). Interestingly, amidst this suppressed immune/ECM phenotype, our data suggest that certain programmed cell death (PDCD) proteins, specifically PDCD10 and PDCD4, display lower levels in the stroma of our patient relative to non-recurrent cases (FDR < 0.05 for difference) (Supplementary Data Fig. S5). Reduced expression of these pro-apoptotic factors might suggest a stromal context that is somewhat permissive to cell survival and possibly responsive to immune checkpoint inhibition (since an immune-reactive environment often correlates with the presence of apoptotic and cytotoxic activity). This observation raises the question of whether the patient’s tumor might have been amenable to immunotherapy, indeed she did receive checkpoint inhibitors, which had to be halted due to toxicity rather than lack of effect. It’s conceivable that her tumor microenvironment might have had some capacity for immune engagement (as hinted by PDCD markers), but unfortunately, the patient could not tolerate the immunotherapy long enough to derive benefit. We also noted an upregulation of ribosomal proteins and protein synthesis machinery in the patient’s stroma (relative to non-recurrent stroma). Heightened ribosomal activity could indicate increased protein production supporting tumor growth and remodeling, another feature consistent with a pro-tumor microenvironment.

All together, these findings suggest that the interplay between the tumor and its stromal microenvironment in our patient was skewed toward a state associated with aggressiveness and recurrence. The primary tumor stroma exhibited molecular hallmarks similar to those of patients who experienced melanoma relapse, including suppressed immune pathways and extracellular matrix alteration. This “pro-metastatic” stromal signature likely contributed to the tumor’s ability to progress and metastasize. From a clinical perspective, the stromal profile might serve as an additional indicator of risk; in the future, patients whose tumors display such stromal features might benefit from closer monitoring or adjuvant therapies aimed at modifying the tumor microenvironment (for example, treatments to boost local immune activity or normalize the stroma). In this case, the stromal analysis reinforces the need for a multi-faceted treatment approach addressing not only the tumor cells but also the supporting microenvironment that can facilitate tumor spread.

## Discussion

This case report illustrates how integrating AI-based digital pathology with spatially resolved proteomics can yield clinically relevant insights into tumor heterogeneity and disease progression in a young patient with advanced melanoma. We developed and applied an innovative pipeline combining deep learning–guided histopathological analysis with laser microdissection and high-resolution proteomics. This approach enabled us to examine the patient’s tumor at multiple levels, identifying distinct subpopulations of melanoma cells in situ, profiling their molecular differences, and relating those differences to the patient’s clinical course and therapeutic responses. By overlaying morphological and molecular data on the timeline of the patient’s disease, we gained a clearer understanding of which tumor cell subset was driving metastasis and how the tumor was adapting at the biochemical level over time. Importantly, this integrated analysis pointed toward possible therapeutic strategies tailored to the tumor’s unique profile, underscoring the translational potential of such an approach in real-world clinical decision-making.

A key finding of our study was the AI model’s ability to discern two spatially defined subclones (PT1 and PT2) within the primary tumor, and to predict that one of them (PT1) had morphological characteristics mirroring the tumor cells in the patient’s metastases. This morphologic prediction was confirmed by the proteomic data: PT1’s protein expression profile clustered closely with the profiles of the lung and brain metastases, whereas PT2’s profile was quite distinct (Fig. 3A). These results demonstrate the power of AI-guided pathology to identify heterogeneity and metastatic-prone subclones within a primary tumor at the time of diagnosis. In traditional practice, a pathologist examining an H&E slide might recognize heterogeneity but could not definitively know which subpopulation, if any, is destined to cause metastases. Here, by training an AI on subtle histological cues and then validating with molecular data, we could retrospectively pinpoint the subclone that drove dissemination. For patients and clinicians, such information could be invaluable: if an aggressive subclone like PT1 is identified early, it might justify more intensive systemic therapy or closer follow-up even if the disease is technically at an earlier stage.

Our combined analysis also provided a deep characterization of the tumor’s biology that helps explain its clinical behavior. Both the PT1 subclone and the metastatic lesions shared a metabolic and signaling signature marked by upregulated glycolysis and mitochondrial OXPHOS, along with activation of the MAPK pathway and other growth pathways (AKT/mTOR). Metabolic plasticity, the ability to switch between or concurrently use glycolytic and oxidative metabolism, has been increasingly recognized as a hallmark of aggressive cancers, including melanoma^12,30–32^. It can confer survival advantages under various conditions (such as hypoxia or fluctuating nutrient supply) and has been linked to therapy resistance and recurrence. In our patient’s tumor, the dual activation of energy pathways suggests the melanoma cells were well-equipped to meet the energetic and biosynthetic demands of rapid growth and metastatic colonization. Consistently, previous studies have shown that high oxidative phosphorylation activity in melanoma is associated with increased risk of recurrence and may drive metastasis, while glycolysis upregulation supports growth in oxygen-poor niches^12,32^. Our findings reinforce those concepts in a concrete clinical scenario.

The enrichment of MAPK pathway activity in the metastases provides molecular support for the use of BRAF/MEK inhibitors in this case, which the patient indeed received during her treatment course. However, our data also hinted at why that targeted therapy alone was insufficient to durably control her disease. We detected elevated levels of SRC and YES1 kinases in the metastases (Supplementary Data Fig. S4), and these are known to reactivate ERK signaling through bypass mechanisms even when BRAF is inhibited^18,19^. This suggests that, although MAPK-targeted treatments likely had some effect (the patient’s disease was stable for a period on BRAF/MEK therapy), the tumor was predisposed to develop resistance via alternate pathways. Clinically, this aligns with the pattern that the patient relapsed relatively soon after completing the adjuvant targeted therapy. It raises the possibility that a combination therapy approach from the outset might have been more effective, for instance, concurrently targeting the parallel pathways or survival strategies the tumor could use. Our analysis strongly supports adding a metabolic inhibitor as one such strategy. Given the extensive metabolic reprogramming observed, targeting mitochondrial function or glycolysis could potentially cripple the tumor’s adaptive resilience. We highlighted metformin as a promising candidate in this regard, due to its capacity to inhibit mitochondrial complex I and its track record of safety^20,21,33^. Notably, metformin’s potential adjuvant benefit in melanoma and other cancers has been reported in multiple studies^22–24^. While metformin is not a traditional anticancer drug, its ability to perturb tumor metabolism and modulate the tumor microenvironment may enhance the effectiveness of immunotherapies and kinase inhibitors. In our patient’s case, one could hypothesize that combining metformin with BRAF/MEK inhibition might have delayed or reduced metastatic recurrence by exploiting the tumor’s metabolic vulnerabilities, an approach that could be tested in future clinical trials for high-risk melanoma patients.

In addition to metabolic modulation, our drug connectivity analysis pointed to EGFR/ERBB inhibitors and mTOR/PI3K inhibitors as potential components of a multi-pronged therapy. While EGFR inhibitors alone have modest efficacy in melanoma^25^, there is a sound rationale for their use in combination regimens to overcome resistance mechanisms^28,29^. Pelitinib, identified in our analysis, is particularly interesting because it irreversibly inhibits EGFR and has shown synergy with BRAF inhibitors in preclinical melanoma models^28^. Given that EGFR and related pathways can crosstalk with the MAPK pathway and that our patient’s tumor expressed proteins that can activate downstream ERK (like SRC), an EGFR inhibitor might have curtailed some of that bypass signaling. Similarly, the upregulation of the AKT/mTOR pathway in the tumor supports the idea of adding an mTOR inhibitor (such as temsirolimus, which our analysis also flagged) to suppress that survival pathway. In practice, combination therapies need to be chosen carefully to manage toxicity, but our omics-driven predictions align with combination strategies that are already under investigation or in use (e.g., MEK inhibitors with PI3K/mTOR inhibitors, or BRAF/MEK with EGFR inhibitors). The take-home message is that precision profiling of the tumor can highlight specific combinations of treatments that might be more effective than any monotherapy, especially in cases where the tumor exhibits multiple concurrent drivers of growth.

Our study also underscores the role of the tumor microenvironment, particularly the stroma, in melanoma progression. The proteomic profile of our patient’s primary tumor stroma closely resembled that of other patients who experienced recurrence, characterized by downregulation of immune response pathways and extracellular matrix components^12^. This kind of immunosuppressive, pro-tumor stroma likely facilitated the melanoma’s aggressive behavior. Interestingly, we noted low levels of PDCD4/PDCD10 in the patient’s stroma, which might indicate an environment that could respond to immune checkpoint inhibitors (since these proteins are related to cell death, and their low expression might correlate with an active immune response if unleashed). However, in our patient, the use of immunotherapy was unfortunately limited by severe toxicity. This highlights a real challenge in oncology: sometimes the theoretically right therapy (e.g., checkpoint blockade in a patient who might benefit from immune activation) cannot be given or continued due to adverse events. It underpins the importance of having alternative strategies; for example, targeting the tumor’s metabolism or other vulnerabilities, as we identified, when standard therapies are not tolerated or fail. The stromal analysis also raises the possibility that targeting the microenvironment (for instance, using therapies to boost immune infiltration or modifying the ECM) could be an adjunct to directly targeting the tumor cells. In melanoma, approaches like adding immune adjuvants or anti-fibrotic agents are being explored to convert an immune-cold tumor into an immune-responsive one. In future cases resembling ours, profiling the stroma might inform such adjunctive measures.

**Fig. 6:**
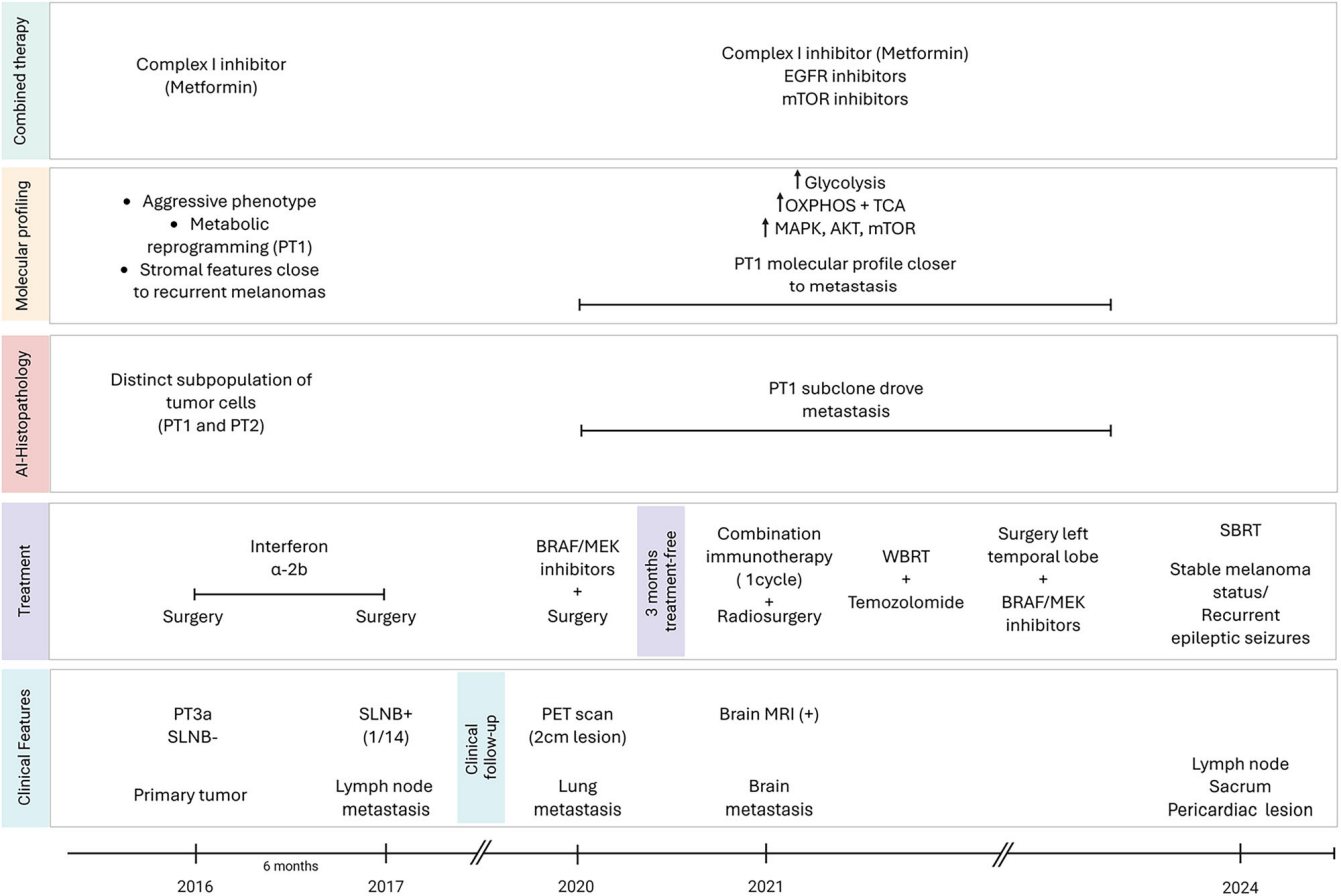
Summary of study findings and patient disease timeline. Schematic overview of the patient’s clinical course, integrating key AI-assisted histological and molecular findings over the progression of the disease. The timeline includes treatments administered and highlights therapeutic strategies suggested based on the patient’s individualized molecular profile.

Figure 6 provides a visual summary of our patient’s journey, integrating the clinical timeline with key findings from the AI and proteomic analyses, and indicating how these insights could correlate with treatment decisions. From a clinical standpoint, the ability to identify an aggressive subclone at diagnosis (via AI histopathology) could prompt earlier systemic therapy or closer monitoring for metastasis in an otherwise young, early-stage patient. Later in the disease, when standard therapies either fail or cannot be tolerated, the detailed molecular information from spatial proteomics can guide the selection of second-line or experimental treatments tailored to the patient’s tumor profile. In our case, had these analyses been available in real time, they might have suggested introducing a metabolic inhibitor or an EGFR/PI3K pathway blocker alongside standard therapy to preempt resistance, hypotheses that now warrant testing in prospective studies or clinical trials.

This study has limitations that should be acknowledged. Here, we applied a patient-specific AI-based digital pathology approach to characterize the morphological features of the individual case under analysis, aiming to maximize classification accuracy within the tissue sample of interest (i.e., primary tumor and metastases). Therefore, applying this workflow to a new case would require retraining. Future work to improve inter-patient generalizability in a larger, more diverse melanoma cohort is currently underway^34^. In this regard, incorporating pathology foundation models pre-trained on large-scale datasets may represent a promising approach, given the transferability capacity these models have demonstrated^35,36^.

Also, it is important to note that while tumor heterogeneity observed between PT1 and PT2 is supported by differential proteomic signatures and morphological parameters such as nuclear size, a formal quantification of spatial complexity was not performed in this study. Entropy-based metrics^37^, represent a promising complementary framework that could be applied to segmented cell data and spatially resolved proteomic profiles to determine indices of tumor heterogeneity. Future work should explore the application of such measures to characterize the structural complexity in tumor samples. Additionally, given this is a single-patient analysis with a limited amount of sample, proteomics and histopathological results are presented as exploratory and hypothesis-generating, and should be interpreted with caution.

Despite the limitations, this case study demonstrates that integrating AI-assisted histopathology with spatial proteomics can provide insights into tumor behavior and uncover therapeutic strategies in a challenging melanoma scenario. By combining detailed tissue image analysis with deep molecular profiling, we were able to trace the patient’s disease progression over time, identify the potential tumor subpopulation responsible for metastasis, and explore potential mechanisms of treatment resistance and sensitivity. These findings set the stage for bringing omics analysis into routine clinical practice, where it can enhance risk stratification, improve predictions of treatment response, and potentially guide the design of combination therapies to extend patient survival. In young patients with aggressive cancer (and in situations where conventional therapies are limited by resistance or toxicity) such a precision oncology approach may offer a new approach to tailor treatments to the unique biology of an individual’s tumor, ultimately improving outcomes.

## Methods

### Sample description

Five formalin-fixed paraffin-embedded (FFPE) tissue blocks were utilized, including one from the primary tumor, three from lung metastases, and one from a brain metastasis. Proteomics profiling of the whole tissue was conducted on all five blocks. For laser microdissection analysis, 10 µm sections were prepared using PEN MembraneSlides (Leica) and stained with H&E. Three blocks were selected: the primary tumor, the lung metastasis with the highest tumor content, and the brain metastasis.

In this study, technical replicates (mass spectrometry injection replicates) were not included. For the brain metastasis sample, laser capture microdissection was performed across two spatially distinct regions within the tissue, yielding two samples that were analyzed separately as biological replicates by nLC-MS/MS. Two spatially distinct regions of interest from the primary tumor were collected, corresponding to the distinct tumor subpopulations PT1 and PT2, and were analyzed separately as individual samples. Whole-tissue analysis of the lung metastasis was performed in triplicate, corresponding to the three available FFPE blocks.

The study workflow was approved by the Hungarian Ministry of Human Resources, Department of Health Administration, and Deputy State Secretary for National Chief Medical Officer (Approval number: 26370-13/2021/EÜIG, and BM/17784-1/2024). The study was conducted in accordance with relevant guidelines and regulations from the Swedish biobanking laws and the Declaration of Helsinki. The patient provided written informed consent for participation and publication of the study.

### Artificial intelligence-based on digital pathology analysis

The primary tumor and two metastases (*i.e*., lung and brain) were analyzed using an AI-based digital pathology approach. The H&E-stained sections were digitized using a Pannoramic 1000 digital scanner (3DHISTECH Ltd., Hungary) at 40× optical magnification (NA: 0.95). The image resolution was 0.12 μm per pixel. Subpopulation of melanoma cells, stroma areas, including tumor-infiltrating lymphocytes (TILs), and normal histological components were identified, and further isolated by laser microdissection.

The single-cell-based AI model was created using deep-learning algorithms to annotate and differentiate the tumor subpopulation of cells (when presented) and stroma content from the primary melanoma and metastasis. Biological Image Analysis software (BIAS, v. 1.4., Single-Cell Technologies) was used for integrative image analysis, which combined image pre-processing, deep-learning-based image segmentation, and deep-learning approaches for tissue part categorization.

In the primary tumor, we segmented the nuclei on the sample using the nucleazer model^9^, dilation (15 micron radius) around the nuclei mask to generate masks that separate the tissue based on the closest nucleus, allowing us to generate contours that can be used later for microdissection. For phenotype classification, a ResNet 50-based classifier model was trained to sort the cells into one of the classes. In this study, the model used for cell phenotype classification was trained and validated on image regions from the same patient’s tissue to maximize classification accuracy within the tissue samples of interest (i.e., primary tumor and metastases). For each cell, a 384-pixel area was cropped and then resized to 224 pixels for input into the ResNet model, with the nucleus centered in the crop. The sample size was augmented 10-fold using random transformations, including cropping, mirroring, rotating, and adjustments to intensity and contrast. A fisheye transformation was applied to the crops to enlarge the center of each cell (factor 1.5 in BIAS settings)^10^. The nucleus mask was also provided as a fourth alpha channel, in addition to the standard Red, Green, and Blue channels, to further direct the model’s attention toward the nucleus region of each crop. The model was trained for 5 epochs and subsequently validated. The number of epochs was determined empirically by analyzing the loss curve until a plateau was observed in the later epochs. Validation was performed by randomly splitting the dataset into 15% validation and 85% training subsets. The original model’s training and validation sets were defined per group as follows: 2697 PT1 cells (2282 training / 415 validation), 1184 PT2 cells (1028 training / 156 validation), 1451 lymphocytes (1227 training / 224 validation), 3248 other cells (dermis, epidermis, adipose, gland — 2732 training / 516 validation), and 2670 segmentation artifacts (2294 training / 376 validation). No additional class balancing techniques were applied. Training was performed with a batch size of 40 and a momentum of 0.9. Brain and lung metastasis samples were analyzed using the same pipeline, trained classifier, and validation settings. Three experts labeled and revised the metastatic tumor areas, and the original skin model was applied against this dataset to identify the subpopulation of cells most closely correlated with the metastatic tumor cells.

The regions selected for laser microdissection in the primary tumor and metastases (lung and brain) were defined based on the AI model output, subsequently validated by expert pathologists, to ensure that only morphologically confirmed tumor and stroma areas (with and without lymphocyte infiltration) were collected. Ambiguous regions were excluded from dissection.

### Laser-microdissection: subpopulation of tumor cells and stroma isolation

Two distinct cell subpopulations (PT1 and PT2) within the primary tumor, as well as tumor cells from metastasis and stromal regions, were isolated and collected for quantitative proteomics from stained tissue sections using laser capture microdissection (Leica, LMD 7). Wide-field optics (20x objective) were used for high cutting precision with a range of 40-50 laser cut energy. The isolated material was collected using a laser pulse and a PCR tube. The contours from the clinically relevant regions identified by the AI model (BIAS, BIAS, v. 1.4., Single-Cell Technologies) were transferred to Leica laser microdissection software, version 8.3.1. Approximately 4000 cells were collected per sample (*i.e*., tumor: 4263 ± 1882 cells, stroma: 3615 ± 1960 cells), taking into account the total area collected and the slide thickness.

### Sample preparation for proteomics

Whole tissue sections (WTS) from FFPE blocks were processed following established protocols^16,17^. Briefly, samples were deparaffinized using a 1:50 dilution of EnVision FLEX Target Retrieval Solution (Agilent Dako), incubated at 97 °C for 10 minutes with shaking (500 RPM). This step was followed by centrifugation at 15,000 × *g* for 15 minutes at 4 °C, and the cycle was repeated until all paraffin was cleared. Laser microdissected (LMD) samples did not require deparaffinization. Protein extraction was carried out using 200 μL of extraction buffer (5% SDS, 25 mM DTT, and 100 mM TEAB) for WTS samples and 40 μL for LMD samples. All samples were incubated at 99 °C for 1 hour to promote antigen retrieval. To enhance protein solubilization, samples were then sonicated using a Bioruptor (40 cycles of 15 seconds on/off at 4 °C). Protein concentration in WTS samples was quantified using the Pierce 660 nm Protein Assay (Thermo Scientific), supplemented with the ionic detergent compatibility reagent. Protein digestion was performed using the S-TRAP micro method, with 2 μg of trypsin for WTS (enzyme-to-protein ratio of 1:25) and 1 μg for LMD samples, according to the manufacturer’s instructions. Peptide content was measured with a NanoDrop spectrophotometer (DeNovix DS-11, USA) and submitted for mass spectrometry analysis.

### Mass spectrometry-based analysis

Mass spectrometry analysis was performed using an Ultimate 3000 RSLCnano UPLC system (Dionex) connected to a Q-Exactive HF-X mass spectrometer (Thermo Fisher Scientific, Waltham, MA, USA). Approximately 1 μg of peptides were injected and analyzed by our nLC-MS/MS approach. The liquid chromatography setup included a PepMap C18 trap column (3 μm, 100 Å, 75 μm ID × 2 cm, nanoViper) and an EASY-Spray RSLC C18 analytical column (2 μm, 100 Å, 75 μm ID × 50 cm). Peptides were loaded using 0.1% TFA in water as the loading buffer at a flow rate of 5 μL/min. All separation solvents were obtained from Thermo Fisher Scientific (Waltham, MA, USA). Column temperatures were maintained at 35 °C for the trap and 60 °C for the analytical column. Peptide separation was achieved using a 100-minute non-linear gradient, starting with 4% buffer B and increasing to 26% over 85 minutes, followed by a ramp up to 50% over the next 15 minutes. Data was acquired using a recently implemented variable window data-independent acquisition (DIA) method^38^.

### Data analysis and statistics

Protein identification and quantification from the DIA runs were performed using DIA-NN software in direct mode, with searches conducted against the 2022 human reference proteome from UniProt. A label-free quantification strategy was used, maintaining a 1% false discovery rate (FDR) at the peptide and protein levels. In total, proteins were confidently identified across samples (Supplementary Data Table S4). Data processing was carried out using Perseus (v1.6.14 and v.2.1.5.0), GraphPad Prism 10.5.0, and BigOmics Analytics. Abundance values were log₂-transformed and normalized by median subtraction.

To assess differential protein abundance, a log2 fold change ≥ 1 was used as the threshold for defining a protein as differentially abundant. Only proteins with at least 70% valid values across samples were included in the analysis, and no missing value imputation was applied. Given the limited sample size, results are presented as exploratory and descriptive. For UMAP (Uniform Manifold Approximation and Projection) analysis, only proteins with at least 70% valid values across the cohort were included, and missing values were imputed. Unsupervised hierarchical clustering analysis was performed using the ComplexHeatmap R/Bioconductor package on scaled log-abundance values (z-score) using Euclidean distance^39^.

To identify the biological pathways dysregulated during tumor evolution, 1D- and 2D-enrichment analyses based on a curated set of cancer-related hallmarks were performed on the proteomics dataset using Perseus (v.2.1.5.0). Pathways with a *p*-value < 0.05 were considered significantly enriched. Gene Set Enrichment Analysis (GSEA) was carried out on the normalized dataset using the BigOmics Analytics.

Drug connectivity analysis was performed by integrating our proteomic data with the L1000 Connectivity Map (CMap)^40^ using the BigOmics platform. In this analysis, the log2FC matrix was extracted for all computed proteomic contrasts and mapped to gene-level identifiers for overlap with the L1000 reference database. Contrasts sharing fewer than 20 genes with the L1000 database were excluded. Drugs were assigned to principal drug classes (meta-sets), and only classes containing at least 10 distinct perturbation profiles were retained. Gene ranks were computed for each contrast and each L1000 drug profile, and rank-based Pearson correlation (R) was calculated. Drug set enrichment analysis was then performed for each contrast against the predefined drug meta-sets using the rank correlation matrix. A GSEA normalized enrichment score (NES), along with associated p- and q-values, was computed for each drug, and the top 11 inhibitor compounds (FDR < 0.05) were highlighted. A plot visualizing the mechanism of action (MOA) across the enriched drug profiles was generated using the GSEA normalized enrichment score of each MOA class. Negative NES values indicate that the drug is inversely correlated with the molecular signature of the samples, which reflects a reversal of the disease-associated gene expression pattern and thus represents a potential therapeutic candidate

### Comparative stromal proteome analysis

The non-recurrent and recurrent reference cohort data used for comparative analysis were previously generated and published by our group^12^. In that study, stroma regions from patients with and without recurrence history were analyzed by spatial proteomics using laser microdissection coupled to nLC-MS/MS, following the same sample preparation method and acquisition protocol applied to the current case report. To ensure direct comparability across datasets, all .raw files from the published reference cohort were reanalyzed jointly with the .raw files from the current patient sample in DIA-NN software, enabling a unified deconvolution of the entire dataset. Quality control samples were included in both analytical runs and confirmed consistent nLC-MS/MS instrument performance. Batch effect correction using Singular Value Decomposition (SVD) was subsequently applied to minimize potential batch-driven variability and ensure the robustness of subsequent statistical comparisons.

## Supplementary information

Supplementary Data

Supplementary Data_Table S4

## Data Availability

The raw proteomics data have been deposited in the ProteomeXchange repository and are publicly accessible under the identifier PXD073928. Whole tissue images and the trained model pipeline are available via the following link: Image Data and AI-trained model. Additional data supporting the findings of this study are provided within the manuscript and supplementary files. Any further data or materials may be requested directly from the authors.
