# Supplementary material for: AI-based digital pathology and spatial proteomics enable precision oncology: A case report of recurrent melanoma in a young patient
Source: NPJ Precis Oncol. 2026 Jul 28;10:296. doi: 10.1038/s41698-026-01569-w (PMC13416036; doi:10.1038/s41698-026-01569-w)
Supplement: Supplementary file 1 — Supplementary Data [file 41698_2026_1569_MOESM1_ESM.pdf]

Supplementary Data

Supplementary Figures

Table S1. Whole slide statistics. Average nucleus and cell size.

All predicted cells*	Average nucleus size	Nucleus size standard deviation	Average cell size	Cell size standard deviation	Count
PT1	48.9055	33.0041	154.609	105.565	57641
PT2	33.1526	17.5677	136.565	83.4434	26957
PT1+PT2	43.8858	29.9064	148.859	99.4096	84598
Lymphocytes	27.1295	15.909	93.9885	53.6286	23524
Dermis + Epidermis + Adipose + Gland	34.1364	24.6203	385.417	312.624	63753
Junks	27.8905	31.1106	405.199	298.794	32657

*Detected cells: 171, 875. Average confidence values for the different classes: PT1 (94.86%), PT2 (86.86%), Lymphocytes (89.94%)

Table S2. Whole slide statistics. Average nucleus and cell size. Analysis of cells with a confidence threshold of ≥99%.

99% confidence	Average nucleus size	Nucleus size standard deviation	Average cell size	Cell size standard deviation	Count
PT1	50.5336	26.161	149.451	80.5031	39620
PT2	34.5917	17.7951	125.365	64.1873	9937
PT1+PT2	47.337	25.5225	144.621	78.1043	49557
Lymphocytes	24.8644	13.9084	80.9977	40.6635	12262
Dermis + Epidermis + Adipose + Gland	35.1713	25.9539	468.646	350.723	24324

Table S3. Evaluation of the original skin model on brain and lung metastatic tissues

	**Annotated as**	**Predicted as**		
	**Tumor**	**PT1**	**PT2**	**Other/Unknown**
Brain	916	664 (72.49%)	175 (19.10%)	77 (8.41%)
Lung	1523	1257 (82.53%)	2 (0.13%)	264 (17.33%)

Table S4. Protein groups identified across samples and cell subpopulations

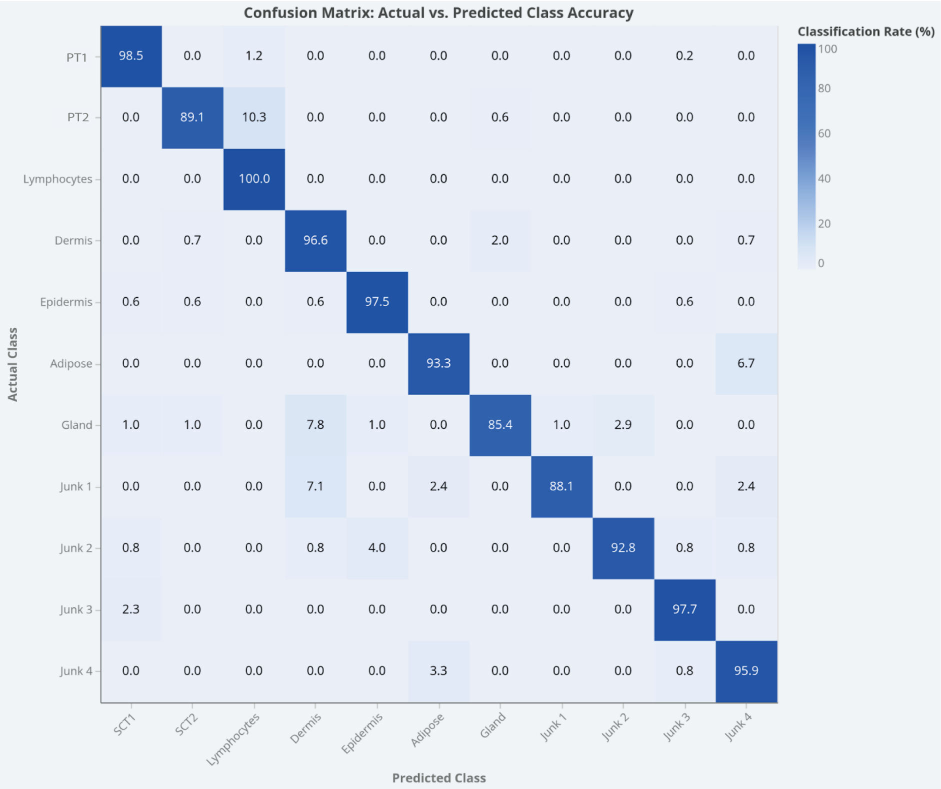

Actual \ Predicted	SCT1	SCT2	Lymphocytes	Dermis	Epidermis	Adipose	Gland	Junk 1	Junk 2	Junk 3	Junk 4
PT1	98.5	0.0	1.2	0.0	0.0	0.0	0.0	0.0	0.0	0.2	0.0
PT2	0.0	89.1	10.3	0.0	0.0	0.0	0.6	0.0	0.0	0.0	0.0
Lymphocytes	0.0	0.0	100.0	0.0	0.0	0.0	0.0	0.0	0.0	0.0	0.0
Dermis	0.0	0.7	0.0	96.6	0.0	0.0	2.0	0.0	0.0	0.0	0.7
Epidermis	0.6	0.6	0.0	0.6	97.5	0.0	0.0	0.0	0.0	0.6	0.0
Adipose	0.0	0.0	0.0	0.0	0.0	93.3	0.0	0.0	0.0	0.0	6.7
Gland	1.0	1.0	0.0	7.8	1.0	0.0	85.4	1.0	2.9	0.0	0.0
Junk 1	0.0	0.0	0.0	7.1	0.0	2.4	0.0	88.1	0.0	0.0	2.4
Junk 2	0.8	0.0	0.0	0.8	4.0	0.0	0.0	0.0	92.8	0.8	0.8
Junk 3	2.3	0.0	0.0	0.0	0.0	0.0	0.0	0.0	0.0	97.7	0.0
Junk 4	0.0	0.0	0.0	0.0	0.0	3.3	0.0	0.0	0.0	0.8	95.9

Figure S1. Confusion matrix of the original skin model validation

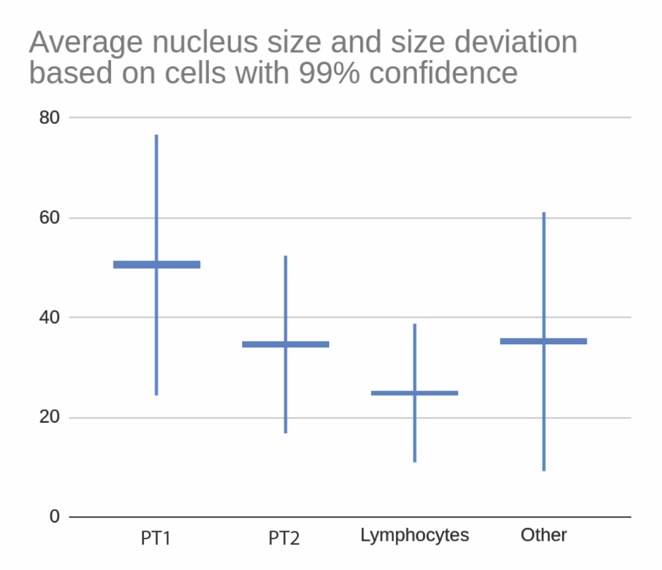

Figure S2. Average nuclear size and the standard deviation for each phenotype classified in the skin tissue section. The other category represents all the remaining cells (*e.g.*, epidermis, dermis, and skin adnexal structures).

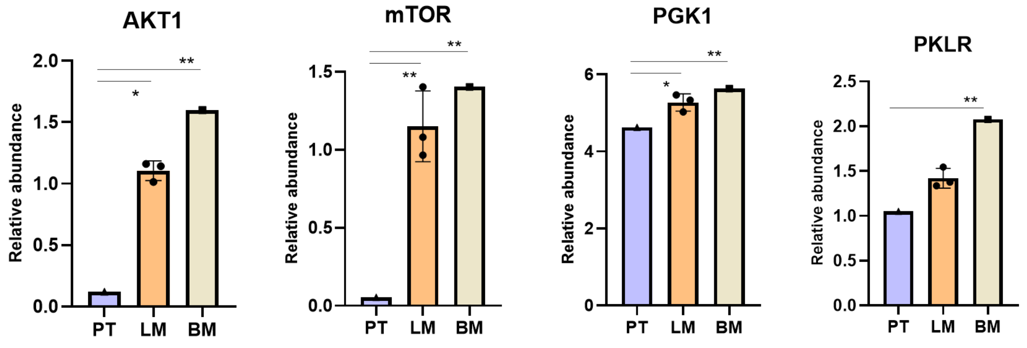

Figure S3. Relative protein abundance of AKT1, mTOR, PGK1, and PKLR in primary and metastatic samples. Whole Tissue analysis. *Log2foldchange>1

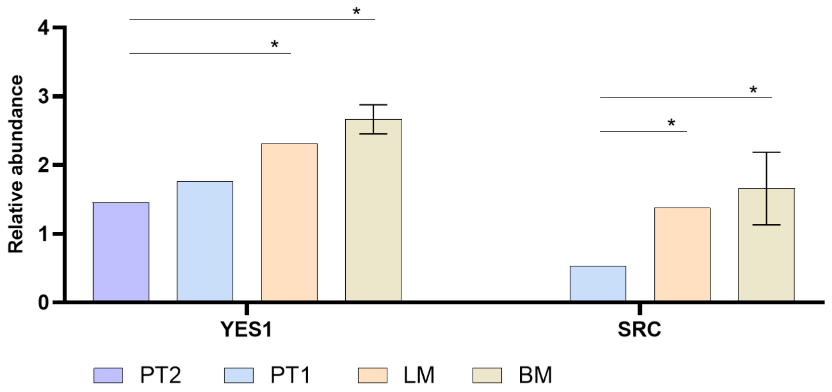

Figure S4. Relative protein abundance of kinases associated with BRAF/MEK inhibitors resistance: YES1 and SRC. *Log2foldchange>1

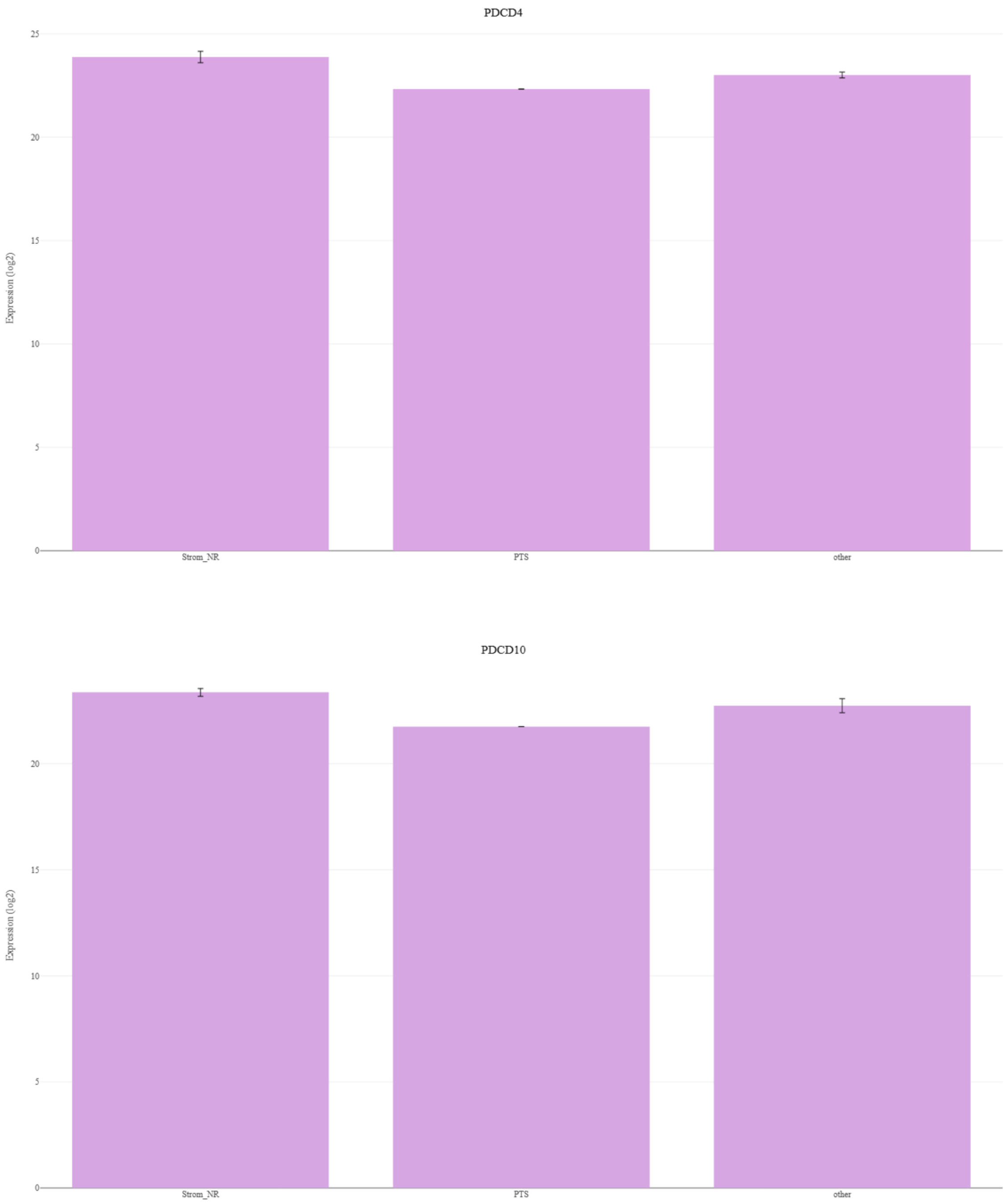

Figure S5. Relative protein abundance of PDCD4 and PDCD10 in the primary tumor stroma compared to stroma from non-recurrent (Stroma_NR) and recurrent melanoma cases (Other).

CARE checklist

	Reporting Item	Page numbers
**Title**	Pipeline description followed by the words "case report"	2
**Keywords**	Five keywords that identify the main topic of the case report.	2
**Abstract**	Pipeline and main findings	2
**Introduction**	• Challenges in metastatic melanoma treatment<br>• Advances in AI-based digital pathology in clinical practice<br>• Integration of AI-based digital pathology and spatial proteomics to enable precision medicine in a challenging melanoma case	2-3
**Results**	• Case presentation: patient information, clinical findings, timeline, diagnostic assessment, therapeutic intervention, follow-up, and outcomes	3-4
	• Highlights of clinical samples and analytical workflow	4-5
	• AI-based digital pathology identifying distinct tumor subpopulations linked to metastasis	5-7
	• Spatial proteomics findings and molecular tumor signatures	7-8
	• Proteomic profiling and insights into off-target treatment strategies	9-11
	• Stromal proteomic profile reflecting an aggressive melanoma microenvironment	12-14
**Discussion**	• Discussion of findings and relevant literature review	14-17
	• Study limitations	17-18
	• "Take away" message of this case report	18
